# Airway epithelial regeneration requires autophagy and glucose metabolism

**DOI:** 10.1038/s41419-019-2111-2

**Published:** 2019-11-20

**Authors:** Kuan Li, Minmin Li, Wenli Li, Hongzhi Yu, Xin Sun, Qiuyang Zhang, Yu Li, Xue Li, Yue Li, E. Dale Abel, Qi Wu, Huaiyong Chen

**Affiliations:** 1 Department of Basic Medicine, Haihe Clinical College of Tianjin Medical University, Tianjin, China; 2 Department of Basic Medicine, Tianjin University Haihe Hospital, Tianjin, China; 3 Fraternal Order of Eagles Diabetes Research Center and Division of Endocrinology and Metabolism, Roy J. and Lucille A. Carver College of Medicine, University of Iowa, Iowa City, IA USA; 4 Key Research Laboratory for Infectious Disease Prevention for State Administration of Traditional Chinese Medicine, Tianjin Institute of Respiratory Diseases, Tianjin, China

**Keywords:** Stem-cell differentiation, Regeneration

## Abstract

Efficient repair of injured epithelium by airway progenitor cells could prevent acute inflammation from progressing into chronic phase in lung. Here, we used small molecules, genetic loss-of-function, organoid cultures, and in vivo lung-injury models to show that autophagy is essential for maintaining the pool of airway stem-like vClub cells by promoting their proliferation during ovalbumin-induced acute inflammation. Mechanistically, impaired autophagy disrupted glucose uptake in vClub progenitor cells, and either reduced accessibility to glucose or partial inhibition of glycolysis promoted the proliferative capacity of vClub progenitor cells and their daughter Club cells. However, glucose deprivation or glycolysis blockade abrogated the proliferative capacity of airway vClub cells and Club cells but promoted ciliated and goblet cell differentiation. Deficiency of glucose transporter-1 suppressed the proliferative capacity of airway progenitor cells after ovalbumin challenge. These findings suggested that autophagy and glucose metabolism are essential for the maintenance of airway epithelium at steady state and during allergic inflammation.

## Introduction

In response to inhaled allergens, acute inflammation is initiated as a host defense; however, asthma develops when early acute inflammation progresses to the chronic and unremitting phase. Asthma is a hard-to-cure disease of the conducting airways that is characterized by airway epithelial damage and remodeling, goblet cell metaplasia, and an influx of eosinophils and neutrophils^1,2^. Tackling the disease during the early acute phase of inflammation represents a potentially promising strategy for curing patients by restoring lung homeostasis.

Airway epithelium plays a critical role in maintaining lung homeostasis^3^. In the previous two decades, much progress has been made on the characterization and functional identification of progenitor cells responsible for airway epithelial repair after injuries, with these findings attributed to the establishment of lineage-tracing transgenic mice and in vitro three-dimensional (3D)-organoid culture models^4,5^. Region-specific epithelial progenitor cells reside in the adult mouse airways^4,6^. As progenitor cells, Club cells (previously known as Clara cells) that express secretoglobin family member 1a (Scgb1a1) and cytochrome P450, family 2, subfamily f, polypeptide 2 (Cyp2f2) are capable of both proliferating and generating differentiated ciliated cells and goblet cells^7,8^. When loss of Club cells occurs (e.g., following naphthalene-induced lung injury), variant Club (vClub, previously called vClara) cells that also express Scgb1a1 in small airways are able to divide and replenish Club cells^9,10^. However, the mechanisms underlying the regulation of proliferation and differentiation of these two epithelial progenitor populations at steady state or during asthmatic inflammation are poorly understood.

Autophagy is a protective lysosome-dependent degradation process of cytoplasmic proteins and organelles by autophagosomes assembled by a series of autophagy-related (*Atg*) genes^11^. This process is activated in response to stress conditions, such as the deprivation of nutrients, including amino acids and glucose^12,13^. AMP activated protein kinase (AMPK) has been shown to trigger autophagy in response to glucose starvation^14,15^. Conversely, autophagy can be abrogated by the mammalian target of rapamycin (mTOR), a central regulator that integrates growth factors and nutrients^16^. Reciprocally, autophagy promotes glucose uptake through glucose transporter-1 (Glut1)^17^. In adult stem/progenitor cells, autophagic activity changes during their differentiation^18,19^. Loss of Atg7 by genetic targeting causes the senescence of satellite cells, which are considered as muscle stem cells^20^, and inhibition of autophagy significantly blocks the proliferation and differentiation of epidermal stem cells, hematopoietic stem cells, and dermal stem cells^18^. Therefore, autophagy appears to play an essential role in maintaining tissue homeostasis; however, it remains unexplored whether epithelial regeneration in conducting airways at steady state or during early acute allergic inflammation is governed by autophagy.

Here, we used immunofluorescence, flow cytometry, genetic loss-of-function experiments, and organoid cultures to investigate the regulation of airway epithelial progenitor cells by autophagy and glucose. We found that autophagy maintained the pool of vClub cells in mouse conducting airways and facilitated glucose uptake in vClub cells. In addition, we demonstrated that glucose withdrawal, glycolysis blockade, or *Glut1* deletion significantly blocked the proliferation of both Club cells and vClub cells, and promoted the differentiation of Club cells into ciliated cells and goblet cells. Furthermore, the levels of autophagy were altered in Club cells and vClub cells during ovalbumin (OVA)-induced acute allergic conditions. These data suggested that autophagy maintains the airway progenitor cell pool by sustaining glucose at a moderate level. Alternation in the levels of autophagy and glucose in airway progenitor cells may not be favorable for the resolution of inflammation associated with airway epithelial damage in chronic phase of asthma.

## Results

### Autophagy maintains the pool of vClub progenitor cells during OVA-induced acute inflammation

**Fig. 1:**
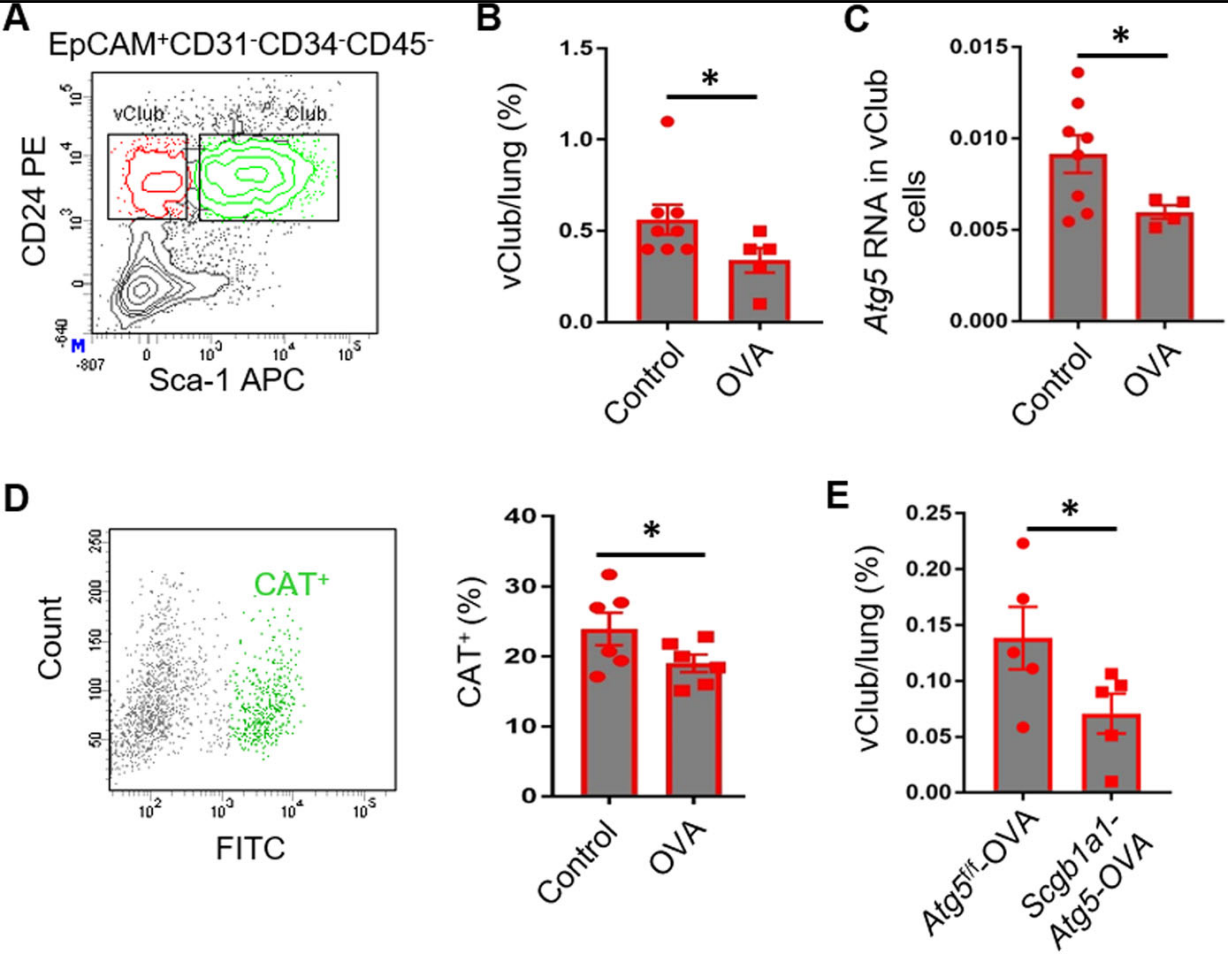
Autophagy maintains the pool of airway vClub progenitor cells during OVA-induced acute inflammation. **a** Sorting strategy of airway epithelial CD24^low^Sca-1^−^ vClub cells (left) and CD24^low^Sca-1^+^ Club cells (right) from mouse lungs by FACS. **b** Quantification of vClub in total lung cells from PBS-treated or OVA-challenged wild type mice by flow cytometric analysis. **c** qPCR analysis of RNA expression of *Atg5* (relative to β-actin) in vClub cells isolated from PBS-treated or OVA-challenged mice. **d** Autophagy induction in airway progenitor cells was analyzed using Cyto-ID labeling (fluorescent cationic amphiphilic tracer CAT) monitored with flow cytometry. **e** Quantification of vClub in total lung cells from tamoxifen-treated *Atg5*^*f/f*^ or *Scgb1a1-Atg5* mice during OVA-induced acute inflammation by flow cytometric analysis. Data represent the mean ± S.E.M. *p < 0.05, Student’s *t* test.

We previously found that mouse airway vClub cells are characterized by epithelial cell-adhesion molecule (EpCAM)^+^CD31^−^CD34^−^CD45^−^ stem cell antigen (Sca)-1^−^CD24^low^ by fluorescence-associated cell sorting (FACS)^4^ (Fig. 1a). Club cells express high levels of Sca-1 as compared with vClub cells (Fig. 1a). Airway epithelial damage is usually observed in the chronic state of human asthma^1^. We therefore determined whether epithelial reparative capacity is abrogated in the airways during the acute phase of inflammation. Following OVA-induced mouse acute allergic inflammation, we observed that the ratio of vClub cells to total lung cells decreased by 20% (Fig. 1b). Genetic variants in *ATG5* have been associated with childhood asthma^21^, and autophagosomes have been observed in bronchial epithelial cells and fibroblasts in asthmatic patients^22^. Here, we observed that during OVA-induced acute inflammation, a significant decrease was observed in *Atg5* expression in vClub cells (Fig. 1c). After OVA challenge, cationic amphiphilic tracer (CAT)-positive fraction was significantly reduced in vClub cells as shown in Cyto-ID Green labeling (Fig. 1d). Although loss of *Atg5* in airway progenitor cells had little effect on the influx of eosinophils, neutrophils, and other inflammatory cells in bronchoalveolar lavage fluid (BALF) or the expression of inflammatory cytokines associated with the recruitment of these inflammatory cells (Figs. S1 and S2), flow analysis indicated that the fraction of vClub cells was significantly decreased in *Scgb1a1-CreER*;*Atg5*^f/f^ (*Scgb1a1-Atg5*) mice relative to that in *Atg5*^*f/f*^ mice during OVA-induced acute inflammation (Fig. 1e). These data suggested that autophagy may be essential for maintaining the pool of mouse airway vClub progenitor cells, although it is not required for the development of OVA-induced acute inflammation.

### Autophagy promotes the colony-forming capacity of vClub progenitor cells

**Fig. 2:**
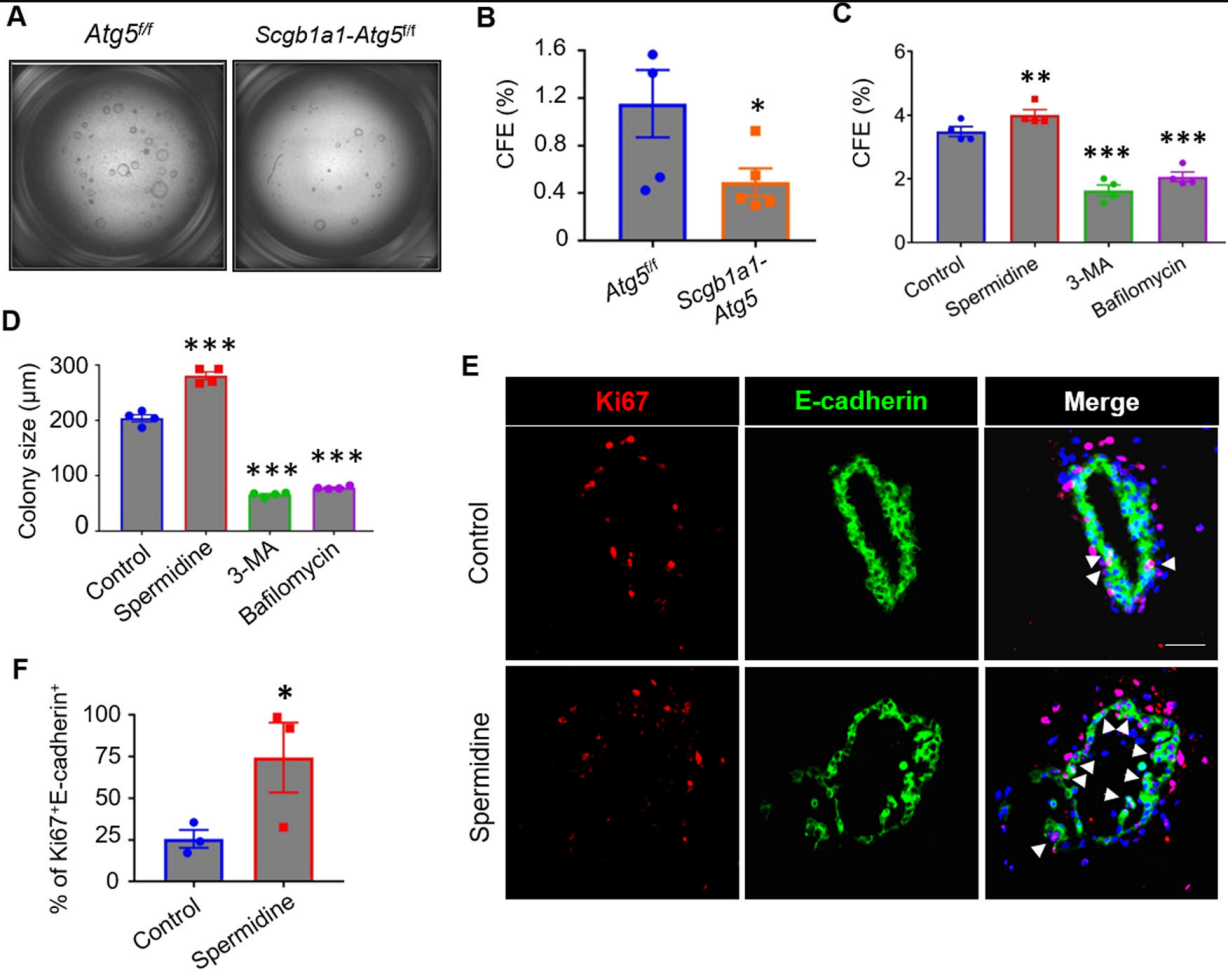
Autophagy promotes the colony-forming ability of vClub progenitor cells. **a** Representative images of Matrigel cultures of vClub cells from tamoxifen-treated *Atg5*^f/f^ or *Scgb1a1*-*Atg5* mice at day 8 after plating. **b** CFEs of vClub cells isolated from tamoxifen-treated *Atg5*^f/f^ or *Scgb1a1*-*Atg5* mice. **c** CFE of vClub cells in the presence of spermidine (1 μM), 3-MA (3 mM) or bafilomycin (10 nM). **d** Diameter of colonies in vClub cell cultures under conditions described in (**c**). **e** Representative images of vClub cell-derived colonies stained with Ki67 (red), E-cadherin (green), and DAPI (blue). Scale bars, 50 μm. Arrowheads indicate Ki67^+^ epithelial cells. **f** Quantification of Ki67^+^E-cadherin^+^ cells in total E-cadherin^+^ cells from vClub cell cultures in the presence of spermidine. Data represent the mean ± S.E.M. *p < 0.05; **p < 0.01; ***p < 0.001, Student’s *t* test.

Next, we investigated the possible role of autophagy in maintenance of the mouse airway epithelium. In vitro 3D organoid culture indicated that *Atg5*^*−/−*^ vClub cells from tamoxifen-treated *Scgb1a1-Atg5* mice displayed significantly lower the colony-forming efficiency (CFE) than vClub cells from *Atg5*^f/f^ littermates (Fig. 2a, b). Consistent with this finding, vClub cells formed markedly smaller and fewer colonies in the presence of the autophagy inhibitors, either 3-MA or bafilomycin (Fig. 2c, d, Fig. S3). Treatment with the autophagy inducer spermidine increased the CFE of vClub cells, as well as the size of the colonies (Fig. 2c, d, Fig. S3). Immunofluorescence staining of epithelial colonies in organoid cultures indicated that the fraction of Ki67^+^E-cadherin^+^ over total E-cadherin^+^ cells was higher in the presence of spermidine (Fig. 2e, f). Together, these data suggested that autophagy is essential for the proliferative capacity of mouse vClub progenitor cells.

### Autophagy enhances vClub progenitor cell differentiation

**Fig. 3:**
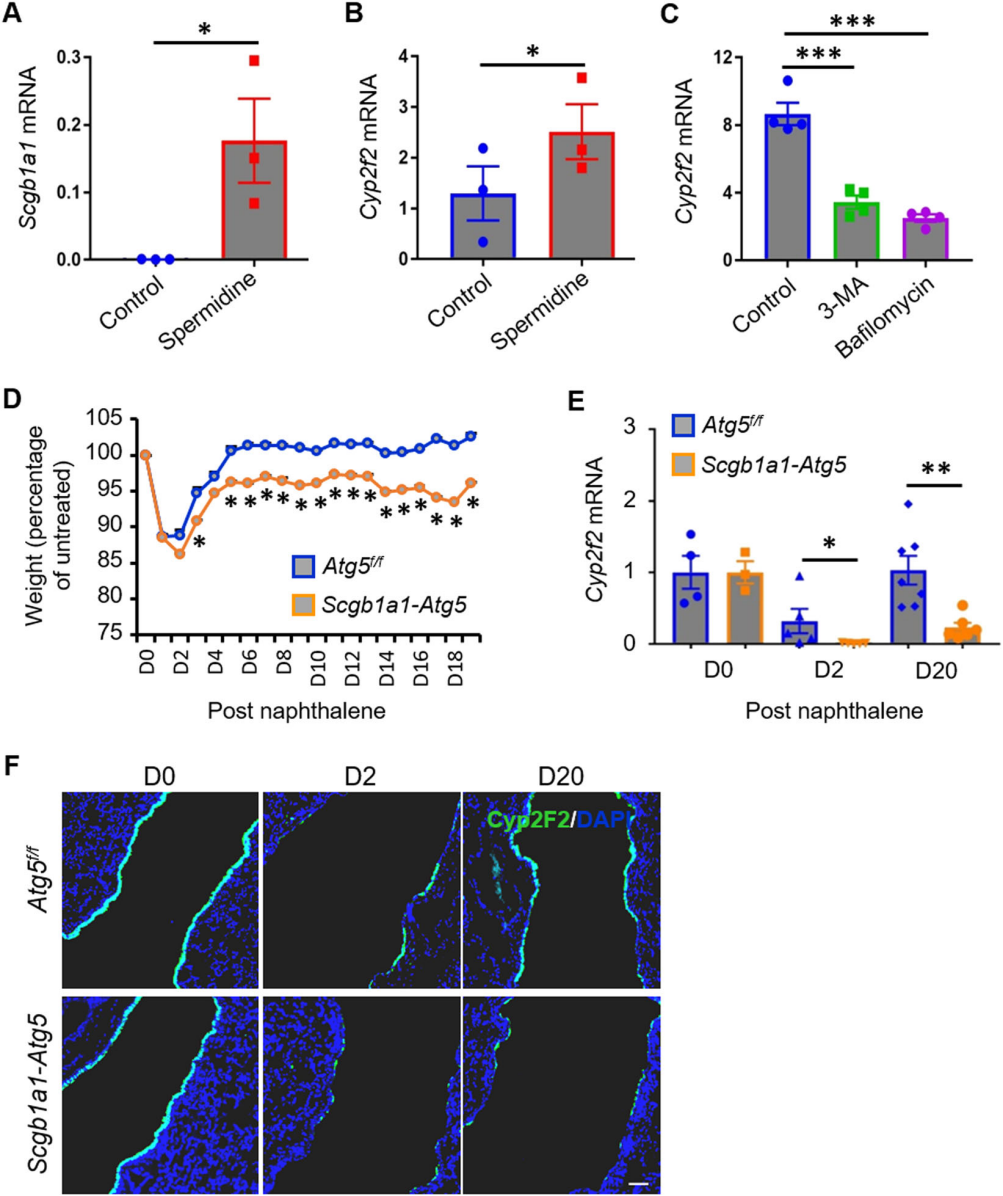
Autophagy enhances the differentiation of vClub cells. **a**, **b** qPCR analysis of *Scgb1a1* and *Cyp2f2* expression (relative to E-cadherin) in organoid cultures of vClub cells in the presence of spermidine at 10 days after seeding. **c** qPCR analysis of *Cyp2f2* expression in organoid cultures of vClub cells in the presence of 3-MA or bafilomycin. **d**, **e** Time course of relative body weight and *Cyp2f2* expression were analyzed in the lungs of tamoxifen-treated *Atg5*^f/f^ or *Scgb1a1*-*Atg5* mice after naphthalene administration. **f** Representative images showing the extent of Club-cell injury or repair in tamoxifen-treated *Atg5*^f/f^ or *Scgb1a1*-*Atg5* mice after naphthalene administration according to immunofluorescence analysis: Cyp2F2 (green); DAPI (blue). Data represent the mean ± S.E.M. *p < 0.05; **p < 0.01; ***p < 0.001, Student’s *t* test.

We then assessed the role of autophagy in the differentiation of vClub cells into Club cells. Expression of Club-cell markers, including *Scgb1a1* and *Cyp2f2*, was promoted in the presence of spermidine, suggesting that autophagy favors the differentiation of vClub cells into Club cells (Fig. 3a, b). In accordance with this, autophagy inhibition by either 3-MA or bafilomycin significantly reduced *Cyp2f2* expression (Fig. 3c). Naphthalene causes severe depletion of Club cells. As predicted, loss of body weight was observed within 2 days of naphthalene administration to *Atg5*^*f/f*^ mice, with a similar loss but insufficient recovery in *Scgb1a1-Atg5* mice (Fig. 3d). qPCR analysis and immunofluence staining of *Cyp2f2* expression in lung tissue confirmed the extensive loss of Club cells at 2 days after naphthalene administration in *Atg5*^*f/f*^ mice, with an even greater loss observed when autophagy was defective in *Scgb1a1-Atg5* mice after tamoxifen treatment (Fig. 3e, f). In addition, recovery of *Cyp2f2* expression was also inefficient in *Scgb1a1-Atg5* mice than in *Atg5*^*f/f*^ mice (Fig. 3e, f). Collectively, these data suggested that autophagy is essential for the generation of Club cells by vClub progenitor cells.

### Distinct role of autophagy in the colony-forming ability and differentiation of Club cells

**Fig. 4:**
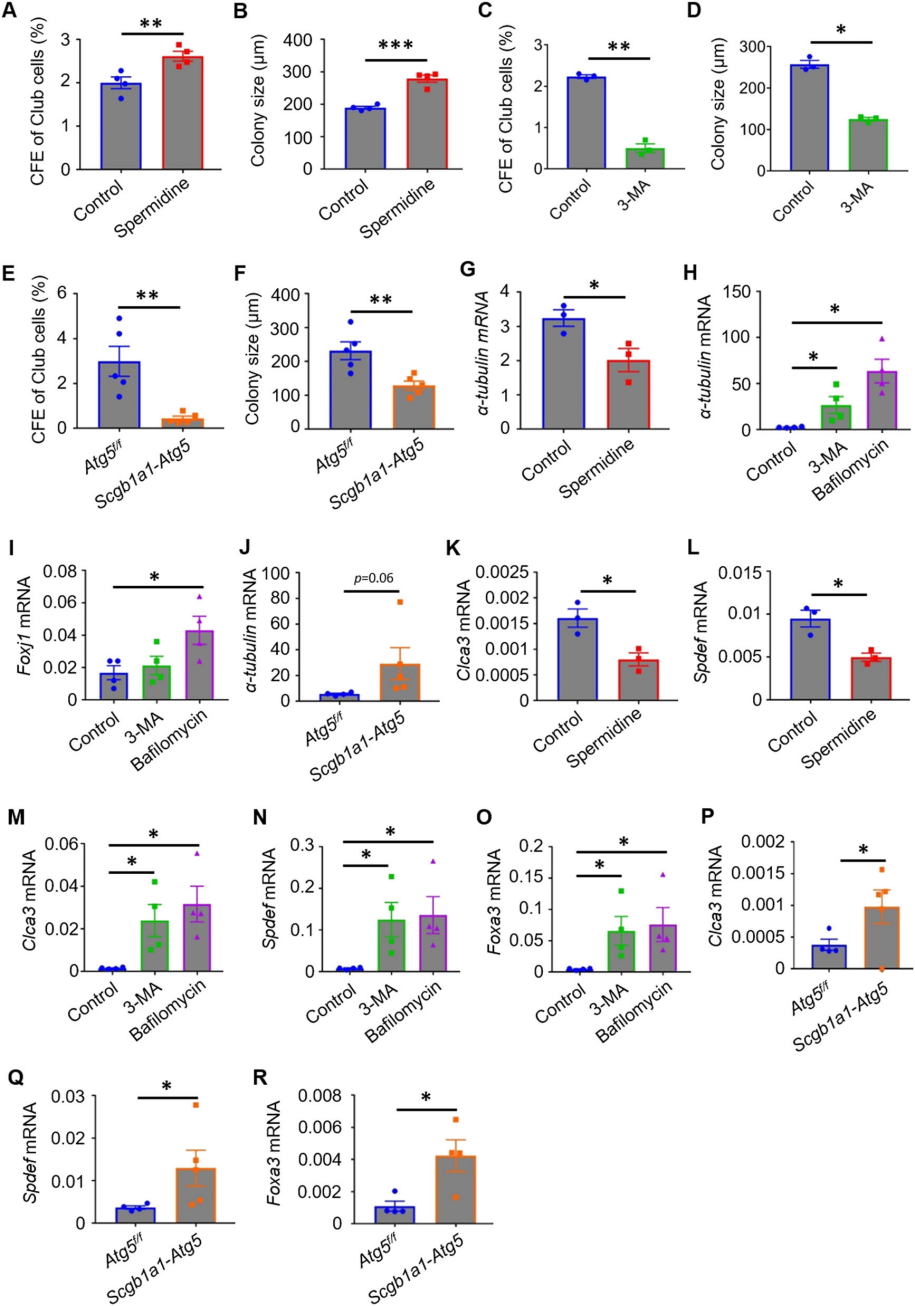
Autophagy facilitates the colony-forming ability, but suppresses the differentiation, of Club cells. **a**–**d** CFEs and mean diameters of epithelial organoids in Club-cell cultures in the presence of spermidine or 3-MA at day 8 after seeding. **e**, **f** CFEs and mean diameters of epithelial organoids in cultures of Club cells isolated from tamoxifen-treated *Atg5*^f/f^ or *Scgb1a1*-*Atg5* mice. **g**–**i** qPCR analysis of the expression of *α-tubulin* and *Foxj1* in organoid cultures of Club cells in the presence of spermidine, 3-MA, or bafilomycin. **j** qPCR analysis of *α-tubulin* expression in organoid cultures of Club cells isolated from tamoxifen-treated *Atg5*^f/f^ or *Scgb1a1*-*Atg5* mice. **k**–**o** qPCR analysis of the expression of *Clca3*, *Spdef*, and *Foxa3* in organoid cultures of Club cells in the presence of spermidine, 3-MA, or bafilomycin. **p**–**r** qPCR analysis of the expression of *Clca3*, *Spdef*, and *Foxa3* in organoid cultures of Club cells isolated from tamoxifen-treated *Atg5*^f/f^ or *Scgb1a1*-*Atg5* mice. E-cadherin was used as the housekeeping gene. Data represent the mean ± S.E.M. *p < 0.05; **p < 0.01; ***p < 0.001, Student’s *t* test.

Club cells are capable of proliferating and differentiating into ciliated cells and goblet cells^7,8^. Therefore, we determined whether the function of Club cells is also governed by autophagy. Club cells, characterized as EpCAM^+^CD24^low^Sca-1^+^ (Fig. 1a) and grown in the presence of spermidine, generated larger colonies and exhibited increased CFEs (Fig. 4a, b). By contrast, those in the presence of 3-MA showed fewer CFEs and formed smaller colonies than those grown in control medium (Fig. 4c, d). *Atg5*^−/−^ Club cells isolated from *Scgb1a1-Atg5* mice after administration of tamoxifen showed markedly lower CFEs and smaller colony size than those from *Atg5*^*f/f*^ littermates (Fig. 4e, f). These data suggested that autophagy promoted the proliferation of Club cells. To evaluate the differentiation of Club cells into ciliated or goblet cells, we analyzed the gene expression of ciliated cell markers including *α-tubulin*, and forkhead box J1 (*Foxj1*), and goblet cell markers including chloride channel, calcium activated, family member 3 (*Clca3*), SAM pointed domain-containing ETS transcription factor (*Spdef*), and forkhead box A3 (Foxa3) in organoid cultures. *α-tubulin* expression was decreased in cultures supplemented with spermidine but increased in cultures with 3-MA or bafilomycin relative to control cultures (Fig. 4g, h). *Foxj1* expression was increased in Club-cell cultures with bafilomycin treatment (Fig. 4i). Similarly, more *α-tubulin* mRNAs were detected in cultures of Club cells isolated from *Scgb1a1-Atg5* mice after tamoxifen treatment than in cultures of those from littermate control mice (Fig. 4j), indicating that autophagy exhibited an inhibitory role in ciliated cell differentiation. Similar to *α-tubulin*, expression of *Clca3, Spdef*, or *Foxa3* was reduced in the presence of spermidine, but was elevated in the presence of 3-MA, bafilomycin, or genetic loss of *Atg5* (Fig. 4k–r). These data suggested that autophagy is essential for the proliferation differentiation of airway progenitor cells.

### Glut1 maintains the pool of airway progenitor cells

**Fig. 5:**
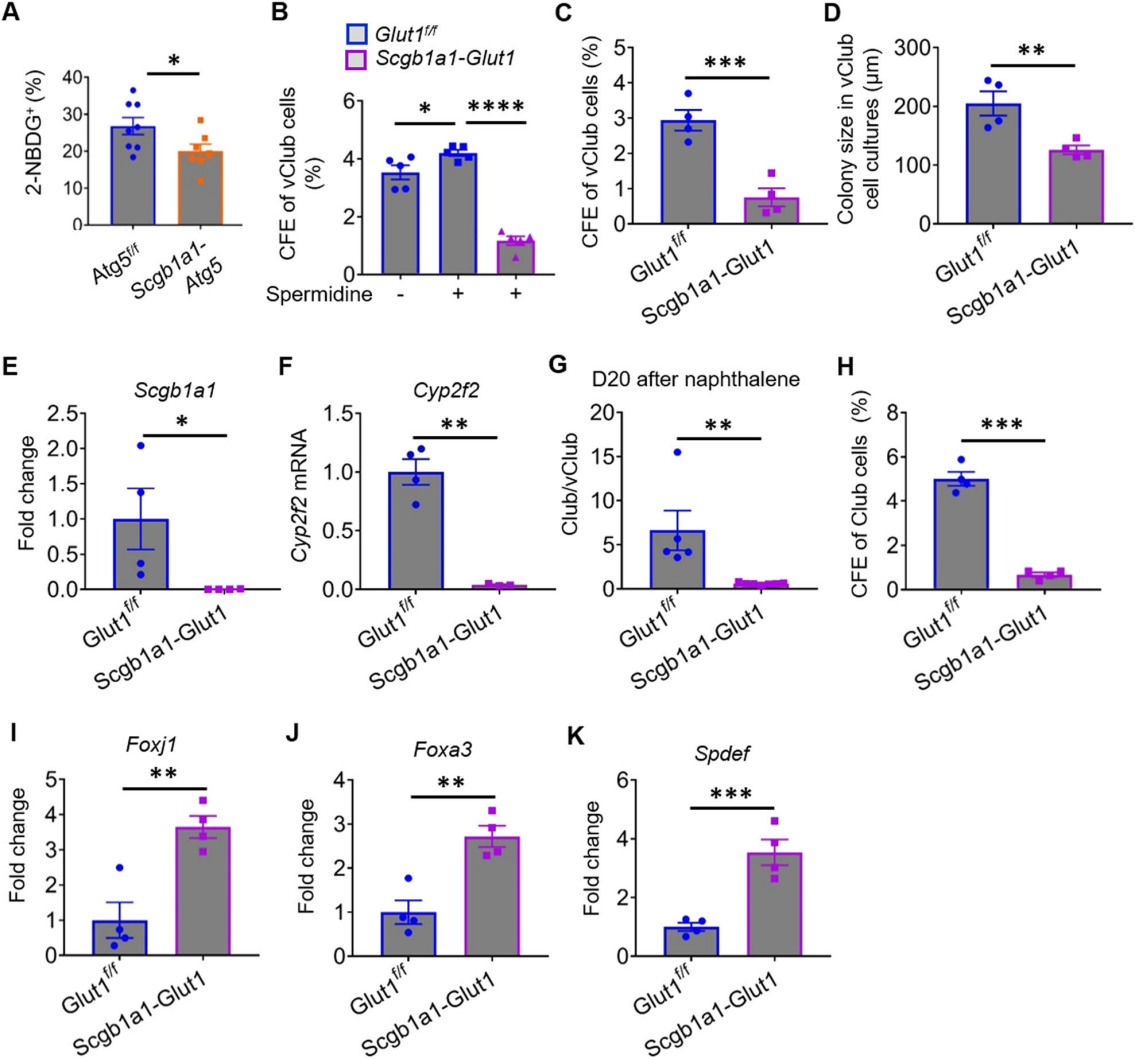
Atg5 loss inhibits glucose uptake by airway vClub progenitor cells. **a** The fraction of 2-NBDG-positive vClub cells (taking up glucose) in tamoxifen-treated *Atg5*^f/f^ or *Scgb1a1*-*Atg5* mice by flow cytometric analysis. **b** CFEs of vClub cells sorted from *Glut1*^*f/f*^ mice in the absence or presence of spermidine and of vClub cells sorted from tamoxifen-treated *Scgb1a1-Glut1* mice. **c**, **d** CFEs and mean diameters of epithelial organoids in Matrigel cultures of vClub cells isolated from tamoxifen-treated *Glut1*^f/f^ or *Scgb1a1*-*Glut1* mice. **e**, **f** Fold change of *Scgb1a1* or *Cyp2f2* mRNA expression in organoid cultures of vClub cells sorted from tamoxifen-treated *Glut1*^f/f^ or *Scgb1a1*-*Glut1* mice. **g** Flow cytometric analysis of total lung cells indicated that the ratio of Club cells to vClub cells was lower in tamoxifen-treated *Scgb1a1*-*Glut1* mice than in *Glut1*^f/f^ mice at day 20 after naphthalene administration. **h** CFEs of epithelial organoids in Matrigel cultures of Club cells isolated from tamoxifen-treated *Glut1*^f/f^ or *Scgb1a1*-*Glut1* mice. **i**–**k** qPCR analysis of fold change of expression of *Foxj1*, *Foxa3*, and *Spdef* in organoid cultures of Club cells isolated from tamoxifen-treated *Glut1*^f/f^ or *Scgb1a1*-*Glut1* mice. Data represent the mean ± S.E.M. *p < 0.05; **p < 0.01; ***p < 0.001; ****p < 0.0001, Student’s *t* test.

Glucose deprivation was shown to induce autophagy, which, in turn, upregulates glucose uptake^17,23^. We determined whether autophagy promotes glucose uptake in vClub cells by 2-(N-(7-nitrobenz-2-oxa-1,3-diazol-4-yl)amino)-2-deoxyglucose (2-NBDG)-trafficking analysis. vClub cells assimilated glucose rapidly (Fig. S4a), and flow cytometric analysis indicated that 28.2% of vClub cells were positive for green fluorescent protein (GFP) at 1 h after incubation with 2-NBDG (Fig. S4b). Glucose uptake by *Atg5*^*−/−*^ vClub cells isolated from tamoxifen-treated *Scgb1a1-Atg5* mice was reduced by 30% relative to that of *Atg5*^*+/+*^ vClub cells (Fig. 5a). Next, we sought to examine whether autophagy facilitates glucose uptake by impacting the expression of glucose transporters (GLUTs). Among 12 GLUT family members, we observed that *Glut1* was the most abundant in both lung epithelial cells and purified airway progenitor cells (Fig. S5a). To evaluate the role of Glut1 in maintaining the function of airway progenitor cells, we established *Scgb1a1-CreER;Glut1*^*f/f*^ (*Scgb1a1-Glut1*) mice. As described, spermidine promoted the CFEs of vClub cells; however, this role was abolished in the absence of Glut1 (Fig. 5b). Quantitative PCR and flow cytometric analysis indicated that Glut1 expression was comparable between *Atg5*^*+/+*^ vClub and *Atg5*^*−/−*^ vClub cells isolated from tamoxifen-treated *Atg5*^*f/f*^ and *Scgb1a1-Atg5* mice (Fig. S5b, c). These data suggested that autophagy facilitates glucose uptake in vClub cells by impacting endocytosis and/or recycling of GLUTs rather than increasing their surface expression.

*Glut1*^*−/−*^ vClub cells isolated from tamoxifen-treated *Scgb1a1-Glut1* mice generated fewer and smaller colonies (Fig. 5c, d). Moreover, the expression of Club-cell markers *Scgb1a1* and *Cyp2f2* was significantly reduced in *Glut1*^*−/−*^ vClub cell cultures relative to littermate controls (Fig. 5e, f), suggesting that the differentiation of vClub cells into Club cells was positively regulated by Glut1. Flow cytometric analysis of total disassociated lung cells indicated that the ratio of Club cells to vClub cells was lower in *Scgb1a1-Glut1* mice than in *Glut1*^*f/f*^ mice at day 20 after naphthalene treatment (Fig. 5g). The CFEs of *Glut1*^*−/−*^ Club cells were significantly lower than those of Club cells from littermate control mice (Fig. 5h). Furthermore, the expression of *Foxj1* was induced in organoid cultures of Club cells isolated from *Scgb1a1-Glut1* mice relative to those from littermate controls (Fig. 5i). Expression of *Foxa3* and *Spdef* was elevated in Club-cell cultures in the absence of Glut1 (Fig. 5j, k). Leukocyte recruitment remained unchanged in the absence of Glut1 during OVA-induced acute inflammation (Fig. S6). These data suggested that Glut1 contributes to maintaining the pool of vClub and Club progenitor cells by promoting their proliferation and inhibiting ciliated and goblet cell differentiation.

### Glucose level determines the function of airway progenitor cells

**Fig. 6:**
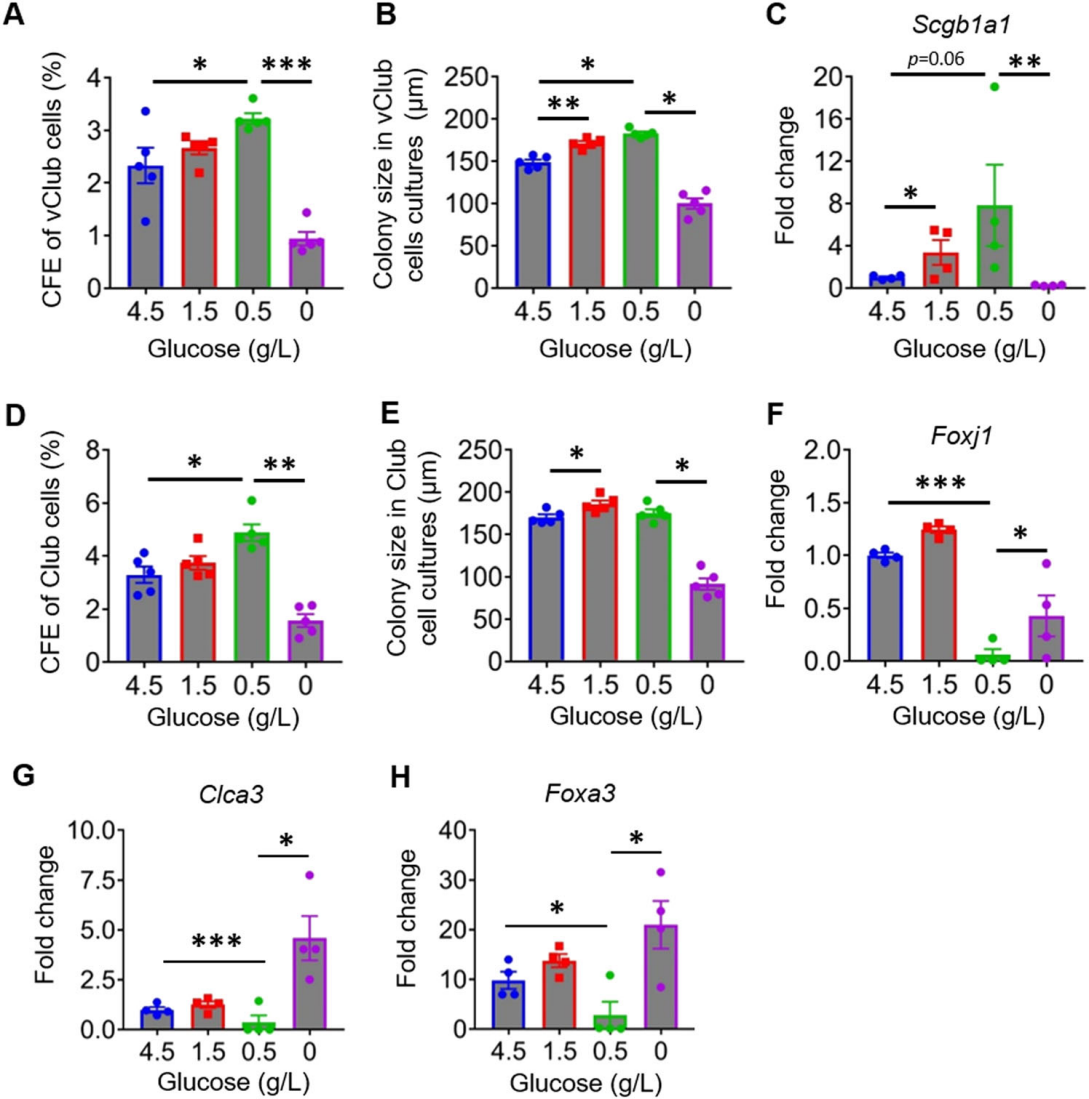
Regulation of airway progenitor cells by glucose. **a**, **b** CFEs and mean diameters of epithelial organoid cultures of vClub cells in the presence of glucose at day 10 after seeding. **c** qPCR analysis of fold change of *Scgb1a1* expression in vClub organoid cultures in the presence of glucose. **d**, **e** CFEs and mean diameters of epithelial organoid cultures of Club cells in the presence of glucose. **f**–**h** qPCR analysis of fold change of *Foxj1*, *Clca3*, and *Foxa3* expression in Club organoid cultures in the presence of glucose. Data represent the mean ± S.E.M. *p < 0.05; **p < 0.01; ***p < 0.001, Student’s *t* test.

We next attempted to explore the regulatory role of glucose in the function of airway progenitor cells. When glucose supplementation decreased, an increase in CFEs and colony size of vClub cells was observed (Fig. 6a, b). However, both CFEs of vClub cells and the size of colonies were significantly reduced in cultures deprived of glucose (Fig. 6a, b). In a similar fashion, *Scgb1a1* expression was promoted in vClub cultures with low amounts of glucose, followed by a significant reduction in cultures without glucose (Fig. 6c). Similarly, both CFEs and colony size were significantly promoted in Club-cell cultures with low amounts of glucose, but were reduced when glucose was deprived (Fig. 6d, e). Expression of *Foxj1* was lower in Club organoid cultures with 0.5 g/L glucose than in the control group (Fig. 6f). Likewise, the expression of *Clca3* and *Foxa3* was also decreased in Club cultures with 0.5 g/L glucose as compared with the control group (4.5 g/L) (Fig. 6g, h). Further deprivation of glucose resulted in a rebound in the expression of *Clca3* and *Foxa3* (Fig. 6g, h). These data indicated that glucose is required for the colony-forming ability and differentiation of mouse airway progenitor cells including vClub cells and Club cells. A decrease in glucose accessibility promotes the colony-forming ability of vClub and Club cells, but inhibits ciliated cell and goblet cell differentiation. Deprivation of glucose may lead to arrested proliferation of airway progenitor cells or even cell death.

### Glycolysis impacts the proliferation and differentiation of airway progenitor cells

**Fig. 7:**
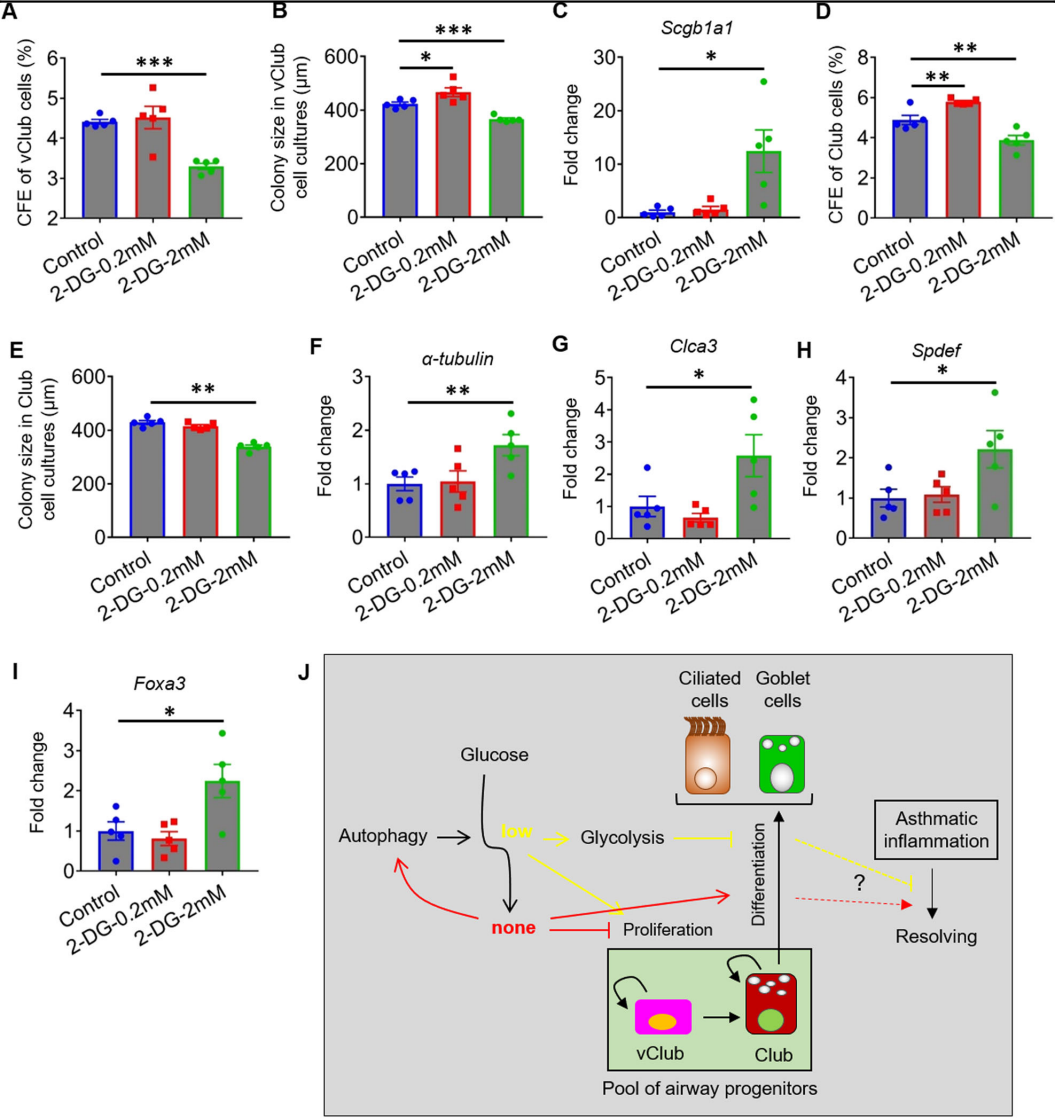
Glycolysis is essential for the function of airway progenitor cells. **a**, **b** CFEs and mean diameters of epithelial organoids in vClub cell cultures with or without 2-DG at day 10 after seeding**. c** qPCR analysis of fold change of *Scgb1a1* expression in organoid cultures of vClub cells in the presence of 2-DG. **d**, **e** CFEs and mean diameters of epithelial organoids in Club-cell cultures with or without 2-DG. **f**–**i** qPCR analysis of fold change of expression of *α-tubulin*, or *Clca3, Spdef*, and *Foxa3* in organoid cultures of Club cells in the presence of 2-DG. Data represent the mean ± S.E.M. *p < 0.05; **p < 0.01; ***p < 0.001, Student’s *t* test. **j** Schematic model describing a possible contribution of autophagy and glucose metabolism to the proliferation and differentiation of airway progenitor cells. Autophagy maintains glucose at low level to sustain the pool of airway progenitor cells. Reduced autophagy in airway progenitor cells may result in insufficient repair of airway epithelia, which is not favorable for the resolution of asthmatic inflammation.

Glycolysis is a series of reactions that extract energy from glucose. We adopted 2-DG to examine glucose metabolism in regulating the function of airway progenitor cells. Mouse vClub cells generated slightly larger colonies, while their CFEs remained unchanged at a lower dose of 2-DG (Fig. 7a, b). However, both CFEs and colony size of vClub cells decreased at a higher dose of 2-DG (Fig. 7a, b). Expression of *Scgb1a1* remained unchanged in vClub cultures at a low dose of 2-DG, but was promoted at a high dose of 2-DG (Fig. 7c). Similar to vClub cells, the CFEs of Club cells were increased at a low dose of 2-DG, but were decreased at a higher dose (Fig. 7d). The size of Club colonies was smaller at a higher dose of 2-DG (Fig. 7e). Expression of *α-tubulin*, *Clca3, Spdef*, and *Foxa3* remained unchanged at a low dose of 2-DG, but was significantly promoted at a higher dose (Fig. 7f–i). This was consistent with our previous findings that the proliferative potential of both vClub and Club cells was promoted at a lower glucose dose, but was inhibited when glucose was totally deprived (Fig. 6). These data suggested that the proliferation of vClub and Club cells are impacted by glycolysis in a two-way fashion.

Together, our data suggested that autophagy is essential for maintaining the pool of airway progenitor cells through sustaining glucose at a moderate level. Low level glucose or partial inhibition of glycolysis promotes the proliferation of both vClub and Club progenitor cells, and suppressing goblet cell differentiation. This may, in part, contribute to the resolving outcome during acute phase of asthmatic inflammation. Blockade of glucose uptake or complete inhibition of glycolysis may lead to a diminished pool of airway progenitor cells through inhibition and acceleration of their proliferation and differentiation, respectively. Eventually, failure to restore airway epithelia may not be favorable for the resolution of asthmatic inflammation in the chronic phase.

## Discussion

Alterations in airway epithelium are related to both the acute and chronic phases of asthma. Obstacles related to asthma treatment are attributable to insufficient understanding of the molecular mechanisms that regulate the pool of airway progenitor cells involved in repopulating the airway epithelium. A major goal of this study was to elucidate the asthma-related role of autophagy in the proliferation and differentiation of airway progenitor cells during tissue homeostasis and OVA-induced acute inflammation. Our main finding was that autophagy maintained the pool of airway Club cells and vClub cells by promoting their proliferation and inhibiting ciliated cell differentiation. Mechanistically, we observed that glucose uptake was facilitated by autophagy in airway vClub progenitor cells and that glucose deprivation, glycolysis inhibition, or Glut1 loss led to a diminished pool of airway progenitor cells by inhibiting and accelerating their proliferation and differentiation, respectively. A moderate level of glucose or glycolysis favors the maintenance of the pool of airway progenitor cells, which may be beneficial for resolving OVA-induced acute inflammation.

Our in vitro 3D organoid experiments demonstrated that induction of autophagy promoted the colony-forming ability of both vClub cells and Club cells in the airway epithelium. Inhibition of autophagy using selective inhibitors or *Atg5* conditional-knockout mice favored the differentiation of Club cells into ciliated cells and goblet cells; this suggested that autophagy is active in maintaining the homeostasis of the slow-turnover epithelium, such as that found in the lung at steady state. Autophagy is usually considered a cellular process associated with stress conditions^24^. Here, we observed that autophagy was protective during naphthalene-induced lung injury.

Autophagy appears to play differential roles in epithelial injury and repair in different organs. Loss of leucine-rich repeat-containing G-protein coupled receptor-5-expressing intestinal stem cells and impairment of epithelial recovery after irradiation were observed in mice lacking *Atg5*^25^. In addition, *Atg5* deletion in keratin-5-positive epithelial cells does not affect the morphology of the thymus, but disturbs the differentiation of the preputial gland in mice^26^. Moreover, Atg16L1 in the intestinal epithelium is critical for preventing the loss of Paneth cells and protecting against exacerbated intestinal injury after murine norovirus infection^27^. Furthermore, autophagy in the proximal, but not distal, tubule epithelium protects against acute kidney injury^28,29^. However, it remains unknown whether autophagy favors epithelial regeneration in the kidney at steady state and after injury, although some types of endogenous stem cells have been proposed^30–32^.

Autophagy in inflammatory cells is also involved in OVA-induced acute inflammation. Mice with deletion of *Atg5* in CD11c^+^ dendritic cells spontaneously develop severe neutrophilic lung inflammation and airway hyperreactivity^33^, suggesting that promoting autophagy in dendritic cells is also critical for controlling asthmatic inflammation. However, a recent study suggested that autophagy is enhanced in B cells by IL4 in the lungs of asthma-prone mice, and that autophagy deficiency in B cells attenuates asthmatic symptoms^34^. We observed that autophagy in airway epithelial progenitor cells maintains the pool of these cells. Insufficient repair of airway epithelium is not favorable for the resolving of inflammation associated with airway epithelial damage in asthma. But this effect may not be observable until the chronic phase of inflammation as loss of Atg5 or Glut1 has little role in the recruitment of inflammatory cells in acute phase. The findings indicate that the contributions of autophagy to asthmatic inflammation are cell-specific; therefore, selective induction of autophagy in epithelial and dendritic cells might be a potential solution.

Genetic variations in ATG5 are associated with childhood asthma^21^, and genetic polymorphisms in ATG5 and ATG7 contribute to neutrophilic airway inflammation in adult asthma^35^. Double-membrane autophagosomes were detected in fibroblasts and epithelial cells in bronchial biopsy tissue from asthma subjects^22^; however, additional information is needed to adequately evaluate the contribution of autophagy in fibroblasts to asthma pathogenesis. Here, we observed that basal level of autophagy in vClub cells was higher than that in Club cells. Although increased autophagy levels were observed in total lung tissue during OVA-induced acute inflammation, autophagy was reduced in vClub cells to a level that was similar to that in Club cells. Possible induction of autophagy in Club cells during OVA-induced acute inflammation sustained the pool of airway progenitor cells by inhibiting the differentiation of ciliated cells and goblet cells. Loss of Atg5 resulted in reduced uptake of glucose in airway progenitor cells; while low accessibility to glucose favored the proliferation of airway progenitor cells. Our data suggested that autophagy is induced when glucose is exhausted; whether proliferative potential of airway progenitor cells is eventually promoted may also be determined by the function of supportive fibroblast cells.

Autophagy is capable of reprogramming cellular metabolism in multiple tissues. A previous study showed that either pharmacological inhibition of autophagy with 3-MA or *Atg5* knockdown significantly increased triglyceride content in hepatocytes^36^. In addition, autophagy maintains the stemness and regenerative potential of hematopoietic stem cells by removing activated mitochondria and controlling oxidative metabolism^37^. Moreover, autophagy facilitates glucose uptake through two pathways in mouse embryonic fibroblasts (i.e., promoting cell-surface expression of Glut1 and enabling the endocytosis and exocytosis of Glut1 trafficking)^17^. In the present study, we discovered that glucose uptake by airway epithelial progenitor cells was promoted by autophagy, possibly through regulating the endocytosis and/or exocytosis of Glut1 trafficking, given that Glut1 expression was not altered in the absence of autophagy. At steady state, deprivation of glucose, inhibition of glycolysis, or knockdown of *Glut1* expression showed that a moderate level of glucose metabolism was critical for the proliferation and differentiation of airway epithelial progenitor cells, which enter a differentiated state when glucose metabolism is blocked. During OVA-induced acute inflammation, *Glut1* expression in airway progenitor cells remained unchanged, although autophagy was altered. However, we cannot rule out the possibility that glucose uptake by airway progenitor cells might be adjusted through other GLUTs during OVA-induced acute inflammation.

In summary, these data suggested autophagy is essential for regenerative capacity of airway progenitor cells. A moderate level of glucose or partial inhibition of glycolysis is beneficial for maintaining airway progenitor cells. However, complete blockade of glucose uptake or glycolysis largely abrogated proliferation but promoted goblet cell differentiation. Failure to restore airway epithelia may accelerate the progression into the chronic state during asthma. Therefore, future investigations are needed to determine whether such progression can be prevented by sustaining glucose at a moderate level in airway progenitor cells, and whether the ability of glucose uptake is abrogated in chronic phase of asthma given that high glucose levels are usually observed in BALF from asthmatic patients^38^.

## Materials and methods

### Reagents

Reagents and their sources are available in the online version of the paper.

### Animal models

C57BL/6 mice were purchased from Huafukang Bioscience Co. Inc. (Beijing, China). *Scgb1a1-CreER* mice were purchased from The Jackson Laboratory (Bar Harbor, ME, USA). *Atg5*^f/f^ mice were provided by the RIKEN BRC through the National Bio-Resource Project of the MEXT (Tokyo, Japan). *Scgb1a1-CreER;Atg5*^f/f^ (*Scgb1a1-Atg5*) mice were created by crossing *Scgb1a1-CreER* mice with *Atg5*^f/f^. *Glut1*^f/f^ mice, generated as previously described^39^, were crossed with *Scgb1a1-CreER* mice to generate *Scgb1a1-CreER;Glut1*^f/f^ (*Scgb1a1-Glut1*) mice. Mice were randomly grouped and age- and sex-matched. Eight- to twelve-week-old mice of both sexes were used in experiments involving OVA-induced acute lung inflammation. All mice were housed in a specific pathogen-free facility at Haihe Clinic College of Tianjin Medical University. All mice were used in accordance with the guidelines approved by the Institutional Animal Care and Use Committee of the Haihe Clinic College of Tianjin Medical University.

### Cell lines

The mouse lung fibroblast cell line MLg was purchased from ATCC (Manassas, VA, USA). MLg cells were maintained in Dulbecco’s modified Eagle medium (DMEM) supplemented with 10% FBS, 100 IU/mL penicillin, and 100 μg/mL streptomycin at 37 °C and 5% CO_2_.

### Tamoxifen and naphthalene administration

Tamoxifen (Sigma-Aldrich, St. Louis, MO, USA) was prepared in corn oil (Sigma-Aldrich) at 20 mg/mL as a stock solution. *Atg5* deletion in *Scgb1a1-Atg5* mice or *Glut1* deletion in *Scgb1a1-Glut1* mice was induced by intraperitoneal injection of 200 mg/kg tamoxifen (Sigma-Aldrich) every other day up to three times. *Atg5*^*f/f*^ littermates or *Glut1*^*f/f*^ littermates received tamoxifen in the same way. *Scgb1a1-Atg5* mice were administered naphthalene (Sigma-Aldrich) at a dose of 250 mg/kg dissolved in corn oil via intraperitoneal injection. At the indicated time points, lung tissues were collected for histological analysis or for isolating lung cells.

### OVA-induced acute lung inflammation

Mice were sensitized by intraperitoneal injection on days 0 and 7 of 100 μL of 10 mg/mL chicken OVA (Sigma-Aldrich) in phosphate-buffered saline (PBS) emulsified with alum. On days 14, 15, and 16, the mice inhaled aerosolized 1% OVA diluted in PBS for 1 h daily. Matched littermate mice used as controls underwent the same procedures, but with OVA replaced by PBS. The mice were sacrificed 2 days after the final challenge, after which BALF was collected and centrifuged. Cells were spread on slides for Diff-Quick staining (Thermo Fisher Scientific, Waltham, MA, USA) according to manufacturer protocol in order to analyze the abundance of infiltrated leukocytes, including eosinophils, neutrophils, and macrophages. Lung tissue was fixed for further immunostaining or was lysed in TRIzol reagent (Invitrogen, Carlsbad, CA, USA) for subsequent gene-expression or western blot analysis. In some experiments, lung tissue was digested with elastase (Worthington Biochemical Corporation, Lakewood, NJ, USA) to allow the sorting of airway epithelial cells.

### Lung dissociation and flow cytometry

Lung tissue was dissociated as described previously^4^. Briefly, lungs were perfused with 5 mL PBS and digested with 4 U/mL elastase and Dnase I (Sigma-Aldrich) to yield single-cell suspensions for flow cytometry. Lung cells were then resuspended in Hank’s balanced saline solution (HBSS) supplemented with 2% fetal bovine serum (FBS), 100 IU/mL penicillin, 100 μg/mL streptomycin, and 10 mM HEPES. Cells were then incubated with primary antibodies, including anti-CD31-biotin (1:40), anti-CD34-biotin (1:20), anti-CD45-biotin (1:67), anti-CD24-phycoerythrin (PE, 1:25), anti-EpCAM-PE-Cyanine7 (PE-Cy7, 1:100), and Sca-1-allophycocyanin (APC, 1:100), followed by incubation with the secondary antibody streptavidin APC-eFluor 780 (1:100). 7-Amino-actinomycin D was added to discriminate dead cells (1:20). Autophagosome formation was analyzed using a CYTO-ID autophagy detection kit with a cationic amphiphilic tracer (CAT) as a fluorescence reagent (Enzo Life Sciences). Flow cytometric analysis or FACS was performed using a FACS Aria III sorter (BD Biosciences, San Jose, CA, USA) and analyzed using FlowJo software (v10.0.7r2; FlowJo, LLC, Ashland, OR, USA).

### Glucose-uptake assays

For flow cytometric glucose-uptake assays, 1 × 10^4^ cells were resuspended in 100 μL glucose-free medium and incubated for 20 min at 37 °C in a humidified atmosphere with 5% CO_2_. 2-NBDG (100 µL dissolved in glucose-free medium; Life Technologies; Thermo Fisher Scientific) was then added to a final concentration of 50 μM, and cells were incubated for an additional 1 h. Cells were washed three times with HBSS-plus (supplemented with 2% FBS, 10 mM HEPES, 100 IU/mL penicillin, and 100 μg/mL streptomycin) and resuspended in 200 μL of HBSS-plus solution before measuring fluorescence by flow cytometry. For fluorescence-microscope-based glucose-uptake assays, cells were washed three times with HBSS-plus, transferred to 96-well plates (Costar; Corning, Corning, NY, USA), and photographed using an Olympus DP80 fluorescence microscope (Olympus, Tokyo, Japan).

### Organoid culture of airway epithelial progenitor cells

As described previously^4^, mouse EpCAM^+^CD24^low^ Sca-1^−^ airway vClub cells (5 × 10^3^ cells/well) and EpCAM^+^CD24^low^ Sca-1^+^ Club cells (5 × 10^3^ cells/well) were resuspended in 100 μL of Matrigel (BD Pharmingen, San Diego, CA, USA) and basic medium (1:1) in the presence of MLg lung fibroblasts (2 × 10^5^ cells/well). The mixture was then added to 24-well Transwell filter inserts (Greiner Bio-One, Kremsmünster, Austria), and the lower chambers were filled with 410 μL DMEM/F12 (Gibco; Thermo Fisher Scientific) supplemented with 10% FBS, penicillin/streptomycin, insulin/transferrin/selenium (Sigma-Aldrich), and 10 μM SB431542 (Sigma-Aldrich). Cultures were maintained in a humidified 37 °C incubator with 5% CO_2_, and fresh culture medium was replaced every other day. Colonies were visualized with an Olympus DP80 inverted fluorescence microscope (Olympus). Colonies with a diameter ≥ 50 μm were counted, and CFE was determined by the number of colonies in each insert as a percentage of the number of input epithelial cells at day 10 after seeding. For gene-expression analysis, TRIzol (Invitrogen) was added to the Matrigel discs, and RNA was extracted from cultures according to the standard protocol. For paraffin embedding, the Matrigel discs were fixed with formalin at 4 °C overnight and then removed and embedded into paraffin according to the standard protocol. Paraffin-embedded colony discs were then sliced at 5 μm for staining.

### Immunofluorescence staining

Lung tissues were routinely perfused, inflated, and fixed in formalin for 2 h at room temperature. Culture colonies were fixed with 4% paraformaldehyde for 1 h at 4 °C, immobilized in O.C.T. compound (Tissue-Tek; Sakura, Torrance, CA, USA), and frozen with liquid nitrogen. Paraffin sections (5 μm) and cyrosections (6 μm) were used for staining. Antigen retrieval was performed in citric acid (10 mM; pH 6), followed by blocking with 5% BSA in 0.2% Triton-X/PBS for 30 min at room temperature. Primary antibodies were incubated overnight at 4 °C at the indicated dilutions: mouse anti-E-cadherin (1:50; Invitrogen), goat anti-Scgb1a1 (1:200; Santa Cruz Biotechnology, Dallas, TX, USA), mouse anti-CYP2F2 (1:100; Santa Cruz Biotechnology), and rabbit anti-Ki67 (1:200; eBioscience). Fluorochrome-conjugated secondary antibodies (1:200; Invitrogen) were incubated at room temperature for 90 min. After antibody staining, sections were mounted with Fluoromount G containing 4′-6′-diamidino-2-phenylindole (DAPI). Stained sections were imaged using a Leica TCS SP5 confocal microscope (Leica, Wetzlar, Germany). The number of E-cadherin^+^ and Ki67^+^ cells was counted in three or more random 20 × views of each colony section, and the percentage of E-cadherin^+^Ki67^+^ cells in the total E-Cadherin^+^ cell population of each colony section was calculated and averaged.

### RNA extraction and qPCR

Total RNA was extracted from lung tissues, airway epithelial cells, or MLg cells using TRIzol reagent (Invitrogen). For qPCR analysis, 0.2 μg total RNA was used for reverse transcription using M-MLV reverse transcriptase (Promega, Madison, WI, USA). cDNA (2.5 µL) was subjected to real-time PCR using SYBR Select master mix (Applied Biosystems, Foster City, CA, USA) and the Roche LightCycler 96 real-time PCR system (Roche, Basel, Switzerland). The amplifying conditions were as follows: 95 °C for 2 min and 40 cycles of 95 °C for 10 s, 60 °C for 20 s, and 72 °C for 20 s. The relative expression level of each gene was determined against *β-actin* levels in the same sample, and fold change in target-gene expression was calculated using the 2^−ΔΔCt^ method, with *β-actin* used for normalization. Primer sequences were as follows: *β-actin*-F: 5′-GGCCAACCGTGAAAAGATGA-3′; *β-actin*-R: 5′-CAGCCTGGATGGCTACGTACA-3′; *E-cadherin*-F: 5′-CTGCTGCTCCTACTGTTTCTAC-3′; *E-cadherin*-R: 5′-TCTTCTTCTCCACCTCCTTCT-3′; *Scgb1a1*-F: 5′-ATCAGAGTCTGGTTATGT-3′; *Scgb1a1*-R: 5′-ATCCACCAGTCTCTTCAG-3′; *Cyp2f2*-F: 5′-CGACTGCTTCCTCACAAAGA-3′; *Cyp2f2*-R: 5′-GTCATCAGCAGGGTATCCATATT-3′; *Clca3*-F: 5′-GGCATCGTCATCGCCATAG-3′; *Clca3*-R: 5′-CACCATGTCCTTTATGTGTTGAATG-3′; *Spdef*-F: 5′-GACTGTGGAATTCCTGGGGG-3′; *Spdef*-R: 5’-ATTGTGGCAGGAGCAGAGAC-3’; *Foxa3*-F: 5′-CTTGGTGGAGGTTGGGTGAG-3′; *Foxa3*-R: 5′-ACAGGCAGTATTCCCAAGCC-3′; *α-tubulin*-F: 5′-GGTGATGTGGTTCCCAAAGA-3′; *α-tubulin*-R: 5′-GTGGGAGGCTGGTAGTTAATG-3′; *Foxj1*-F: 5′-AGAGAGTGAGGGCAAGAGAC-3′; *Foxj1*-R: 5′-GCGGGCTTAGAGACCATTTC-3′.

### Statistical analysis

Data are representative of at least three independent experimental replicates and expressed as the mean ± S.E.M. *P*-values between the experimental and control groups were calculated using two-tailed unpaired Student’s *t* test. *P*-values (*p*) < 0.05 were considered statistically significant (*p < 0.05; **p < 0.01; ***p < 0.001). Age-matched mice were assigned to control or treatment groups without a method of randomization. Sample size for animal experiments was determined based on pilot experiments. Outside of technical errors, no animals were excluded from the statistical analyses. Data were normally distributed, and no significant variance between groups was observed. Variance was similar between the groups that were being statistically compared.

## Supplementary information

Supplemental Materials

Fig S1

Fig S2

Fig S3

Fig S4

Fig S5

Fig S6

## Data Availability

The authors declare that the main data supporting the findings of this study are available within the article and its Supplementary Information. All other remaining data supporting the findings of this study are available from the authors upon reasonable request.
