# Supplementary material for: Airway epithelial regeneration requires autophagy and glucose metabolism
Source: Cell Death Dis. 2019 Nov 20;10(12):875. doi: 10.1038/s41419-019-2111-2 (PMC6868131; doi:10.1038/s41419-019-2111-2)
Supplement: Supplementary file 1 — Supplemental Materials [file 41419_2019_2111_MOESM1_ESM.docx]

**Supplemental materials**

**Materials and methods**

**Key resources table**

| **REAGENT or RESOURCE** | **SOURCE** | **IDENTIFIER** | |
| --- | --- | --- | --- |
| Antibodies |  |  | |
| CD11b Monoclonal Antibody (M1/70), APC-Cyanine7 | Invitrogen | Cat#: A15390 | |
| PE/Cy7 anti-mouse F4/80 Antibody | BioLegend | Cat#: 123113 | |
| Alexa Fluor® 647 anti-mouse FcεRIα Antibody | BioLegend | Cat#: 134309 | |
| CD117 (c-Kit) Monoclonal Antibody (2B8), PE | eBioscience | Cat#: 12-1171-81 | |
| FITC anti-mouse CD206 (MMR) Antibody | BioLegend | Cat#: 141703 | |
| FITC anti-mouse CD4 Antibody | BioLegend | Cat#: 100405 | |
| IL-17A Monoclonal Antibody (eBio17B7), PE | eBioscience | Cat#: 12-7177-81 | |
| IFN gamma Monoclonal Antibody (XMG1.2), APC | eBioscience | Cat#: 17-7311-81 | |
| FOXP3 Monoclonal Antibody (FJK-16s), PE | eBioscience | Cat#: 12-5773-80 | |
| IL-10 Monoclonal Antibody (JES3-9D7), PE-Cyanine7 | eBioscience | Cat#: 25-7108-41 | |
| APC anti-mouse/human IL-5 Antibody | BioLegend | Cat#: 504305 | |
| PE Rat Anti-Mouse TNF | BD Pharmingen | Cat#: 561063 | |
| APC anti-mouse Ly-6G Antibody | BioLegend | Cat#: 127613 | |
| FITC anti-mouse CD193 (CCR3) Antibody | BioLegend | Cat#: 144510 | |
| Chemicals, Peptides, and Recombinant Proteins | | |  |
| O.C.T. compound | Tissue-Tek | Cat#: 4583 | |
| 3-Methyladenine | Sigma | Cat#: M9281-100mg | |
| Bafilomycin | Sigma | Cat#: B1793 | |
| Spermidine | Sigma | Cat#: S2626-5G | |
| Corn Oil | Sigma | Cat#: C8267-500mL | |
| Tamoxifen | Sigma | Cat#: T5648-1G | |
| Elastase | Worthington Biochemical Corporation | Cat#: LS002279 | |
| Growth factor reduced Matrigel | BD Pharmingen | Cat#: 356231 | |
| Diff Quik Hema 3 Stain | Fisher Scientific | Cat#: 122-911 | |
| Insulin/transferrin/selenium | Sigma | Cat#: I3146 | |
| DMEM/F12 | Gibco | Cat#: 11320-033 | |
| Ethylene glycol-bis (EGTA) | Sigma | Cat#: E8145-10G | |
| Deoxyribonuclease Ⅰ | Sigma | Cat#: DN-25 | |
| Imject Alum Adjuvant | Thermo Scientific | Cat#: 77161 | |
| SB431542 | Sigma | Cat#: S4317 | |
| Penicillin-streptomycin | Gibco | Cat#: 15140-122 | |
| HEPES | Sigma | Cat#: H0087 | |
| Thincert cell culture insert | Greiner Bio-One | Cat#: 662641 | |
| Hank's Balanced Salt Solution (HBSS) | Cellgro | Cat#: 21-023-CV | |
| 2-Deoxy-D-glucose | Sigma | Cat#: D8375-1G | |
| L-Leucrine | Sigma | Cat#: L8912-25G | |
| DMEM/F12 50/50 medium | Corning | Cat#: 16-405-CV | |
| Albumin from chicken egg white (OVA) | Sigma | Cat#: A5503-10g | |
| FBS | Gibco | Cat#: 16000-044 | |
| Red Blood Cell Lysing Buffer | Sigma | Cat#: R7767-100mL | |
| 2-NBDG | Life technologies | Cat#: N13195 | |
| TRIzol^TM^ LS Reagent | Invitrogen | Cat#: 10296010 | |
| M-MLV Reverse Transcriptase | Promega | Cat#: M1705 | |
| SSⅢ | Invitrogen | Cat#: 18080044 | |
| RRI | TaKara | Cat#: 2313A | |
| Radom Primer | TaKara | Cat#: D3801 | |
| SYBR SELECT MASTER MIX | Applied Biosystems | Cat#: 4472908 | |
| Naphthalene | Sigma | Cat#: 147141 | |
| Experimental Models: Organisms/Strains | | |  |
| *Scgb1a1-CreER^TM^* | Jackson Laboratories | Cat#: 016225 | |
| *Atg5^f/f^* | The RIKEN BRC through the National Bio-Resource Project of the MEXT in Japan | N/A | |
| *Glut1^f/f^* | N/A | N/A | |
| Biological Samples: Cell Lines |  |  | |
| MLg2908 | ATCC | CCL-206 | |
| Software and Algorithms |  |  | |
| Flow Jo X 10.0.7r2 | Flow Jo | http://www.flowjo.com/ | |
| ImageJ 1.50i | National Institutes of Health, USA | http://imagej.nih.gov/ij | |

**Intracellular cytokine staining by FACS**

Bronchoalveolar lavage fluid (BALF) was collected from control or OVA-treated mice and centrifuged. Cells were resuspended with FACS wash (PBS with 2% FBS and 5mM EDTA), and incubated with antibodies for 40min, including anti-CD11b-APC-Cyanine7 (1μg/mL), anti-F4/80-PE-Cyanine7 (2μg/mL), anti-FcεRIα-eFluor 647 (5μg/mL), anti-CD117 (c-Kit)-PE (2μg/mL), anti-CD4-FITC (5μg/mL), anti-Ly-6G-APC (2μg/mL), or anti-CCR3-FITC (5μg/mL). For staining for intracellular cytokines, cells were centrifuged and then resuspended with 1% PFA (1% PFA in PBS) for 30min. ICS wash (FACS wash with 0.1% Triton X-100) was added to cells for 10min. Cells were then incubated with antibodies for 45min, including FITC anti-CD206 (MMR)-FITC (5μg/mL), anti-IL-17A-PE (2μg/mL), anti-IFN-γ-APC (2μg/mL), anti-FoxP3-PE (2μg/mL), anti-IL-10-PE-Cyanine7, anti-IL-5-APC (2μg/mL), or anti-TNF-PE (2μg/mL). Cells were analyzed by FACS.

**Primers for qPCR**

*TNFα*-F: 5’-TTGTCTACTCCCAGGTTCTCT-3’

*TNFα*-R: 5’-GAGGTTGACTTTCTCCTGGTATG-3’

*IL-1β*-F: 5’-CCACCTCAATGGACAGAATATCA-3’

*IL-1β*-R: 5’-CCCAAGGCCACAGGTATTT-3’

*IL-10*-F: 5’-TTGAATTCCCTGGGTGAGAAG-3’

*IL-10*-R: 5’-TCCACTGCCTTGCTCTTATTT-3’

*IL-4*-F: 5’-TTGAGAGAGATCATCGGCATTT-3’

*IL-4*-R: 5’-CTCACTCTCTGTGGTGTTCTTC-3’

*IL-5*-F: 5’-AGGATGCTTCTGCACTTGAG-3’

*IL-5*-R: 5’-AGCTGTGTCAAGGTCTCTTTC-3’

*IL-13*-F: 5’-GCAGCATGGTATGGAGTGT-3’

*IL-13*-R: 5’-TATCCTCTGGGTCCTGTAGATG-3’

*FoxP3*-F: 5’-TTTCACCTATGCCACCCTTATC-3’

*FoxP3*-R: 5’-GTAGGCGAACATGCGAGTAA-3’

*IFN-γ*-F: 5’-CTCTTCCTCATGGCTGTTTCT-3’

*IFN-γ*-R: 5’-TTCTTCCACATCTATGCCACTT-3’

*IL-17*-F: 5’-CGCAATGAAGACCCTGATAGAT-3’

*IL-17*-R: 5’-CTCTTGCTGGATGAGAACAGAA-3’

*IL-22*-F: 5’-AGCTTGAGGTGTCCAACTTC-3’

*IL-22*-R: 5’-CCGGACATCTGTGTTGTTATCT-3’

*F4/80*-F: 5’-CGTCAGGTACGGGATGAATATAAG-3’

*F4/80*-R: 5’-ATCTTGGAAGTGGATGGCATAG-3’

*FcεRIα*-F: 5’-TCCTGCTATGGGAACAATCAC-3’

*FcεRIα*-R: 5’-GGCACTCACAATGACCAAATG-3’

*Glut1*-F: 5’-CCTCGTGCTCTTCTTCATCTT-3’;

*Glut1*-R: 5’-CTCGGGTGTCTTGTCACTTT-3’;

*Glut2*-F: 5’-CTTACAGTCACACCAGCATACA -3’;

*Glut2*-R: 5’-AGACAGAGACCAGAGCATAGT-3’;

*Glut3*-F: 5’-CACTTTGGAAGAGCGGTTAGA-3’;

*Glut3*-R: 5’-TCATGCCACCAACAGAGAAG-3’;

*Glut4*-F: 5’-GTTTCTCCAACTGGACCTGTAA-3’;

*Glut4*-R: 5’-GGACGGCAAATAGAAGGAAGA -3’;

*Glut5*-F: 5’-CTGTTTCCTCACCACCATCTAC-3’;

*Glut5*-R: 5’-ACGTCTGACACCTTGTTCTTC-3’;

*Glut6*-F: 5’-CAGCACTACACCTGGACAAA -3’;

*Glut6*-R: 5’-CCAGGAGGTCATTGAGTAACATAG -3’;

*Glut7*-F: 5’-AGGCCGAGATGGAAGAAATG-3’;

*Glut7*-R: 5’-GCCATGAGCACAACGATAGA-3’;

*Glut8*-F: 5’-CTGGTTCATGGCCTTTCTAGTG-3’;

*Glut8*-R: 5’-GGACAACGGTCAGTGTGAATAG -3’

*Glut9*-F: 5’-GGTCTGTGACTGTGTCCATATT-3’;

*Glut9*-R: 5’-GGACTTCCTCCCAAGAAACTT-3’;

*Glut10*-F: 5’-CTTGGACCTTCCTGCTCTATG-3’;

*Glut10*-R: 5’-TGTCTGAAACTGCTGCTCTATT-3’;

*Glut12*-F: 5’-GGCCAGCTTGCTTGTTTATG-3’;

*Glut12*-R: 5’-CCTCTAATTCCACCGGGAAAG-3’;

*Glut13*-F: 5’-GTCACCATCAACACCCTCTT-3’;

*Glut13*-R: 5’-CATGTACCTCCATCCATCCTTC-3’;

**Supplemental figure legends**

**Figure S1. Autophagy in airway progenitors is dispensable for leukocyte recruitment during OVA-induced acute inflammation. a** Bronchoalveolar lavage fluid (BALF) was harvested from *Atg5^f/f^* mice or *Scgb1a1-Atg5^f/f^* mice after OVA challenge. Cells in BALF were counted. **b-i** Analysis of the indicated cell types including TNFα-producing neutrophils, IL10-producing neutrophils, eosinophils, Th1, Th2, Th17, Treg, M1 and M2 macrophages by FACS.

**Figure S2. Autophagy in airway progenitor cells exhibits little role in the expression of immunological markers in the lung during OVA-induced acute inflammation.** Lungs were harvested from *Atg5^f/f^* mice or *Scgb1a1-Atg5^f/f^* mice after OVA challenge. Total RNA was extracted from these lungs for quantitative PCR analysis of indicated genes.

**Figure S3. Colony forming ability of vClub progenitor cells is influenced by autophagy regulators.** Representative images of 3D organoid cultures of vClub cells isolated from wild-type C57BL/6J mice in the presence of spermidine (1 μM), 3-MA (3 mM) or bafilomycin (10 nM) at day 8 after plating. Scale bar, 500 μm.

**Figure S4. Monitor glucose uptake by 2-NBDG. a** Images of the bright and fluorescent fields showing uptake of 2-NBDG by vClub cells sorted from *Atg5^f/f^* mice by FACS. **b** After incubation with 2-NBDG, vClub cells were harvested for flow cytometric analysis. Blue: control group; red: 2-NBDG group.

**Figure S5. Autophagy minimally effects the expression of GLUT family members in vClub and Club cells at steady state.** **a** qPCR analysis of the expression of GLUT members in vClub, Club, EpCAM+ lung epithelial cells, total unsorted lung cells from mice and MLg cells. **b** The expression of GLUT members in vClub and Club cells isolated from tamoxifen-treated *Atg5*^f/f^ or *Scgb1a1*-*Atg5* mice. **c** Surface expression of Glut1 on vClub cells by flow cytometric analysis.

**Figure S6. Glut1 loss in airway progenitor cells is dispensable for leukocyte recruitment during OVA-induced acute inflammation.** Quantification of infiltrated inflammatory cells in BALF from control or OVA-challenged *Atg5^f/f^* mice or tamoxifen-treated *Scgb1a1-Glut1* mice by Diff Quick Hema 3 staining.
