# Supplementary figures and images for: Airway epithelial regeneration requires autophagy and glucose metabolism
Source: Cell Death Dis. 2019 Nov 20;10(12):875. doi: 10.1038/s41419-019-2111-2 (PMC6868131; doi:10.1038/s41419-019-2111-2)

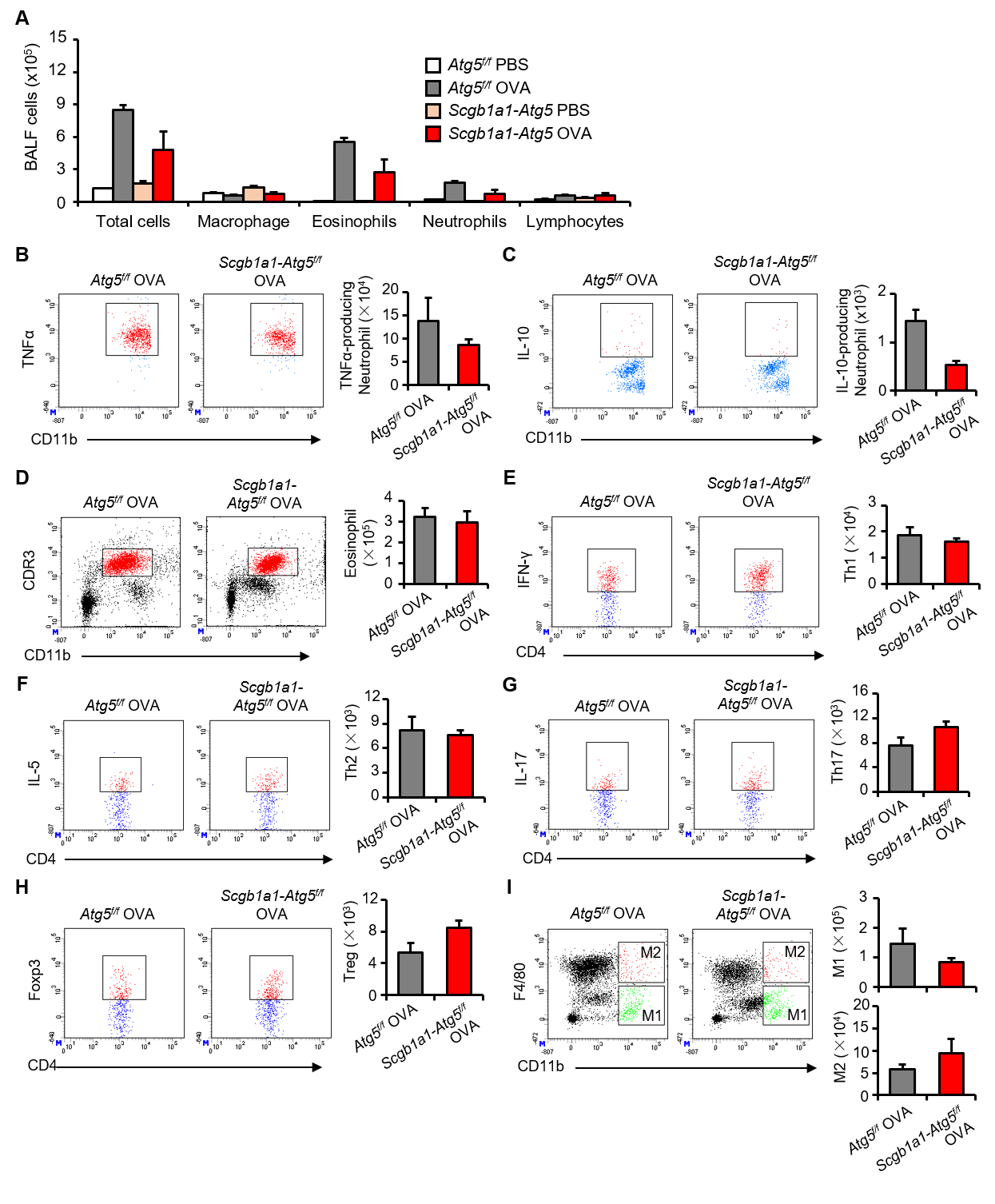

Supplement: Supplementary file 2 — Fig S1 [file 41419_2019_2111_MOESM2_ESM.tif]

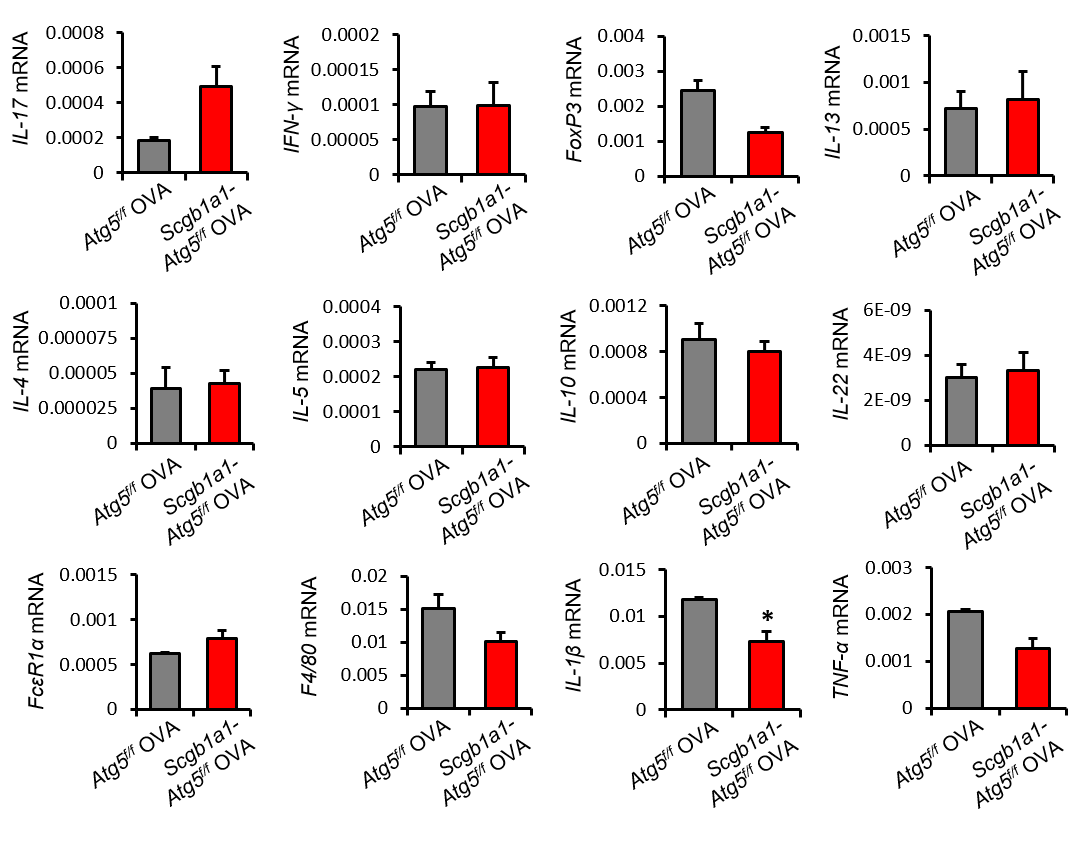

Supplement: Supplementary file 3 — Fig S2 [file 41419_2019_2111_MOESM3_ESM.tif]

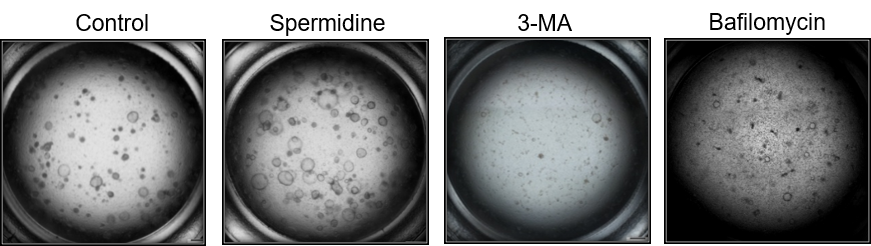

Supplement: Supplementary file 4 — Fig S3 [file 41419_2019_2111_MOESM4_ESM.tif]

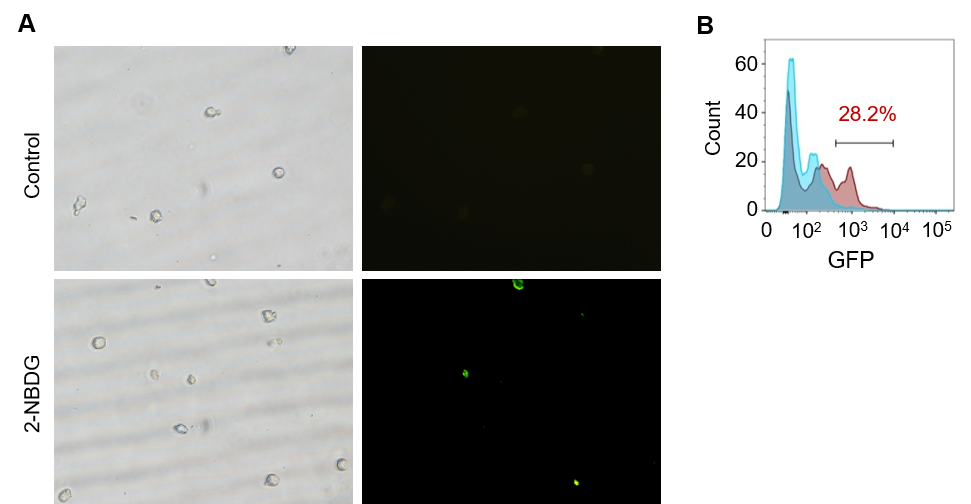

Supplement: Supplementary file 5 — Fig S4 [file 41419_2019_2111_MOESM5_ESM.tif]

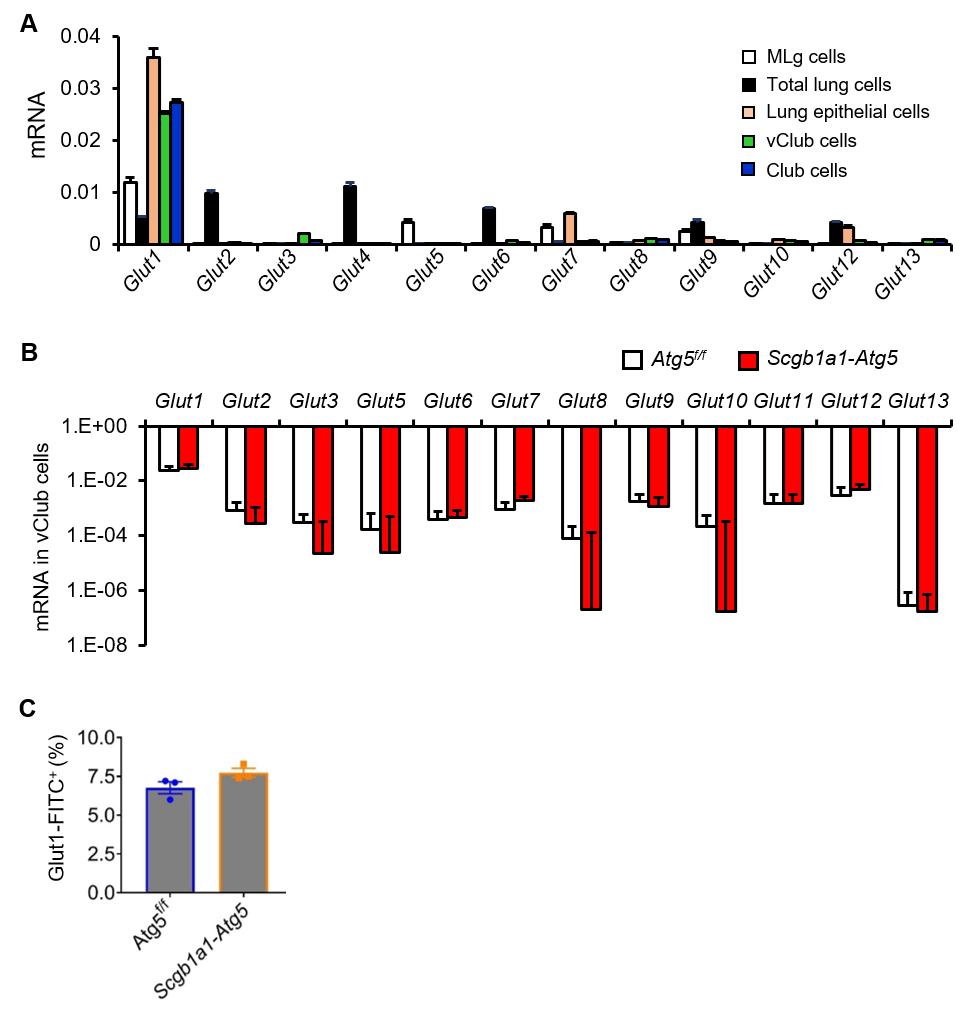

Supplement: Supplementary file 6 — Fig S5 [file 41419_2019_2111_MOESM6_ESM.tif]

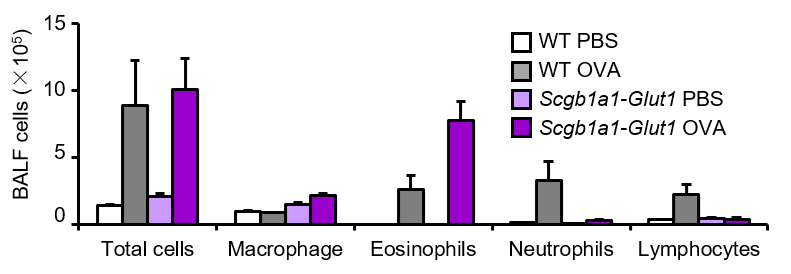

Supplement: Supplementary file 7 — Fig S6 [file 41419_2019_2111_MOESM7_ESM.tif]
